# Toll-Like Receptor Stimulation by MicroRNAs in Acute Graft-vs.-Host Disease

**DOI:** 10.3389/fimmu.2018.02561

**Published:** 2018-11-05

**Authors:** Nina C. Zitzer, Ramiro Garzon, Parvathi Ranganathan

**Affiliations:** ^1^Division of Hematology, Department of Internal Medicine, Comprehensive Cancer Center, The Ohio State University, Columbus, OH, United States; ^2^Department of Veterinary Biosciences, College of Veterinary Medicine, The Ohio State University, Columbus, OH, United States

**Keywords:** graft-vs.-host disease, Toll-like receptors, microRNAs, allogeneic stem cell transplantation, innate immunity

## Abstract

Acute graft-vs.-host disease (aGVHD) is a frequent complication of allogeneic hematopoietic stem cell transplantation (allo-HSCT), accounting for substantial morbidity and mortality associated with this treatment modality. The pathogenesis of aGVHD involves a complex cascade of humoral and cellular interactions in which donor T cells target HLA mismatched host tissues, causing tissue injury through secretion of pro-inflammatory cytokines and induction of direct cytotoxicity. Toll-like receptors (TLRs) are key components of the innate immune system that recognize endogenous danger-associated molecular patterns (DAMPs) and exogenous pathogen-associated molecular patterns (PAMPs). Patients receiving conditioning chemotherapy and/or whole-body irradiation prior to all-HSCT are prone to gastrointestinal damage and translocation of microbiota across compromised intestinal epithelium, resulting in release of PAMPs and DAMPs. These “danger signals” play critical roles in disease pathogenesis by both initiating and propagating aGVHD through dendritic cell maturation and alloreactive T cell responses. There are 10–15 TLRs identified in mammalian species, a subset of which recognize single-stranded RNA (ssRNA) and serve as a key component of viral immunity. Recently, ssRNAs other than those of viral origin have been investigated as potential ligands of TLRs. MicroRNAs (miRs) are short (19–24 nt) non-coding RNAs that play critical roles in a variety of diseases. While traditionally miRs post-translationally modulate gene expression, non-canonical functions such as regulating TLR stimulation by acting as TLR ligands have been described. Here, we review the role of TLRs in aGVHD pathogenesis, the function of miRs in TLR stimulation, and the recent literature describing miRs as TLR ligands in aGVHD.

## Introduction

Acute graft-vs.-host disease (aGVHD) is a frequent complication of allogeneic hematopoietic stem cell transplants (allo-HSCTs), with 30–75% of allo-HSCT recipients developing aGVHD (1, 2). Furthermore, aGVHD accounts for ~10% of all non-relapse mortality in patients that receive allo-HSCT (3, 4), and those with severe aGVHD have a poor prognosis, with an overall 2 years survival of 20–30% (5–7). The morbidity and mortality associated with aGVHD pose a substantial barrier against the wider and safer application of HSCT as a curative modality.

While current prophylaxis and therapeutics function through systemic immunosuppression (1, 8–14), these treatments increase the risk of systemic infections and leukemia relapse (14–16). Therefore, aGVHD research efforts are being focused on not only the development of novel treatment strategies, but more so on the deeper understanding of aGVHD pathogenesis so that aGVHD may be prevented. The potent activation of Toll-like receptors (TLRs) on antigen presenting cells (APCs) following conditioning regimens is often considered a critical initiating event in the development of aGVHD (2, 17–20). Here, we review the current understanding of the classical role of TLRs and their ligands in aGVHD pathogenesis as well as the recent literature describing microRNAs (miRs) as novel ligands for TLRs both broadly and in the context of aGVHD.

## Toll-like receptors (TLRs)

Toll-like receptors are a family of evolutionarily-conserved transmembrane pattern recognition receptors (PRRs) that are critical for innate immune responses and the cross-talk between innate and adaptive immune systems. The concept of PRRs is attributed to Dr. Charles Janeway, who, in 1989, proposed the existence of immune receptors on surveillance cells such as APCs. which allow the innate immune system to specifically recognize microbial infections and mount an appropriate immune response (21). The first member of the Toll family was identified in *Drosophila* flies in 1988, although at that time its function was only recognized as being critical for dorsoventral polarity during fly embryo development (22). The connection between *Drosophila* Toll and innate immunity was not recognized until later, when *Drosophila* Toll and human IL-1R were identified as having homologous cytoplasmic domains and the capability of inducing Rel family transcription factor activation (23). Furthermore, it was observed that *Drosophila* flies that carried non-functional *Toll* genes demonstrated significant defects in antifungal responses, although immune responses to bacterial organisms remained intact (24). In 1997, the first human Toll homolog, called hToll (now known as TLR4), was cloned and was shown to signal through the NF-κB signaling pathway, resulting in the production of inflammatory cytokines during the adaptive immune response (25). One year later in 1998, the connection between TLR4 and its ligand LPS was recognized as endotoxin-tolerant mouse strains were shown to have point mutations in the Tlr4 gene (26, 27). To date, there are 13 TLRs identified between mice and humans which allow the innate immune system to recognize not only bacteria but also viruses, fungi, and protozoa (28, 29).

## TLRs in aGVHD

aGVHD is a complex, multistep disease in which immunocompetent donor T cells destroy MHC-mismatched host tissues by secreting inflammatory cytokines and/or direct cytotoxicity (30, 31). However, pathogenesis of aGVHD is a self-perpetuating cycle that often begins even before the graft is transplanted into the patient. Whole body irradiation and/or chemotherapy frequently used as conditioning regimens are very efficient in reducing leukemia burden and clearing any immune or hematopoietic cells prior to transplantation to prevent graft rejection (32). The cytotoxic effects, however, are not specific to only leukocytes or other hematopoietic cells within the body. Instead, the GI tract is one of the most sensitive organs to radiation and chemotherapy induced acute damage (33, 34–36). Following conditioning therapy, there is extensive tissue damage in the GI as well as compromise to the GI epithelium. This allows translocation of GI flora across the mucosal barrier (37, 38) resulting in the release of inflammatory cytokines (39), danger associated molecular patterns (DAMPs), and pathogen associated molecular patterns (PAMPs). These molecules are then recognized by PRRs on APCs, allowing for their activation (40–42).

DAMPs, also called alarmins, are host-derived “danger” signals produced by the body to allow the immune system to recognize times of extreme cellular stress (43). Typically, the release of DAMPs from damaged tissues occurs when the cells undergoes necrosis (as opposed to apoptosis) since the process of necrosis leads to cell swelling and lysis. DAMPs can arise from two sources in the body: intracellular or extracellular (44). Intracellular DAMPs are released from necrotic cells and include shock proteins (45) and purine metabolites such as ATP (46). On the other hand, extracellular DAMPs arise from breakdown products of the extracellular matrix surrounding stressed cells. Examples of extracellular DAMPs include biglycan, heparin sulfate, and hyaluronan (47). PAMPs, in contrast, are molecules found in/on infectious agents that allow the immune system to recognize exogenous organisms. In aGVHD, PAMPs generally arise from translocated GI flora from the lumen of the intestines to tissues or blood. Examples of common PAMPs critical for aGVHD pathogenesis include lipopolysaccharide (LPS), flagellin, peptidoglycans, and microbial CpG-DNA (17, 48–50). Donor and recipient (host) APCs recognize DAMPs and PAMPs through PRRs, the most well-described of which are TLRs (19, 51). For example, microbial PAMPs such as LPS, flagellin, and CpG-motifs, which may be found in or on translocated GI bacteria, are recognized by TLRs 4, 5, and 9, respectively (48, 50). The consequences of TLR activation in aGVHD are upregulation of adhesion molecules, human leukocyte antigen molecules, and pro-inflammatory cytokine production such as IL-1β, IL-6, IL-12, TNFα, and IFNγ. The downstream effects of TLR-induced APC stimulation are the potent donor T cell activation, expansion, differentiation, and trafficking in aGVHD (52).

As TLRs are important for the initiation of aGVHD, researchers have studied the roles of single nucleotide polymorphisms (SNPs) in TLRs of both donor and recipients and their impacts on aGVHD development. The most well-studied TLR polymorphisms in aGVHD are Asp299Gly and Thr399Ile in TLR4. These TLR4 SNPs were first described in allo-HSCT donors and recipients by Lorenz et al. (53). In this report, the authors demonstrated that the presence of polymorphisms in either the donor or recipient are associated with a lower incidence of aGVHD, although statistical significance was not achieved. Elmaagacli et al. documented slightly conflicting results, with the presence of Thr399Ile SNP in TLR4 in either donor alone or both recipient and donor being associated with more severe aGVHD using univariant analysis; however, statistical significance was lost when using multivariant analysis (54). SNPs in TLR9 have also been shown to impact aGVHD susceptibility, as patients receiving allo-HSCT from donors with either one of two SNPs in TLR9 demonstrated more frequent grade II-IV aGVHD when compared to those receiving allo-HSCT from donors with wildtype TLR9 (55). Sivula et al. demonstrated that many different TLR SNPs found in allo-HSCT donors and/or recipients are associated with aGVHD occurrence when evaluated independently from one another, including one SNP in TLR1, one SNP in TLR4, three SNPs in TLR5, one SNP in TLR6, and one SNP in TLR10, based on multivariant analyses (56). Interestingly, the authors also demonstrate that one SNP in TLR4 found in allo-HSCT recipients was protective from aGVHD.

Yet another focus of research regarding TLRs and aGVHD is how modulation of TLR activation impacts aGVHD development, with the goal of developing novel prophylaxis and therapeutics. Utilizing TLR4 wildtype or deficient mice, Zhao et al. demonstrated that inactivation of TLR4 in either the donor or recipient is protective against aGVHD, with recipient mice having reduced aGVHD symptoms and delayed mortality (57). These finding were also supported by Brennan et al. who showed that administration of heparan sulfate (a TLR4 agonist) promotes aGVHD development while administration of α1-antitrypsin [a serine protease inhibitor which functions as a TLR4 antagonist by disrupting the LPS-TLR4-NF-κB axis (58–60)] reduced aGVHD severity (61). Similar to TLR4, TLR9 inactivation in recipient mice through global deletion, significantly reduces aGVHD severity and mortality (62). Further supporting this data, Taylor et al. demonstrated that repeated administration of CpG oligodeoxynucleotides, a main ligand of TLR9, accelerated aGVHD lethality (63). There are other TLRs such as TLR7 that may accelerate or ameliorate aGVHD depending on time and duration of administration. Chakraverty et al. showed that topical application of R-848, a TLR7/8 agonist, induced severe donor T cell infiltration into the skin of recipient mice 64. Similar results were obtained independently by another research group who repeatedly administered either 3M-011 (a TLR7/8 agonist) or drug vehicle and observed that mice receiving 3M-011 had higher overall mortality when compared to mice receiving vehicle (63). In contrast, from data Jasperson et al. indicated that a single administration of 3M-011 between lethal irradiation conditioning and allo-HSCT induced the tryptophan catabolic pathway in APCs, leading to significantly reduced lethality and colonic pathology scores (65). While the pharmacologic studies described here are all based on well-studied methods of TLR activation or inhibition, researchers are also actively investigating other novel mechanisms by which TLRs are activated and can be pharmacologically manipulated.

## MiRs as TLR ligands

MicroRNAs (miRs) are small, non-coding RNAs that are approximately 19–24 nucleotides long and are found in nearly all plants and animals (66). They function in regulating gene expression for critical cellular processes such as cell development, differentiation, expansion, survival, and function (67). The canonical function of miRs involves the mature miR, loaded into the RISC complex, interacting with target mRNA or proteins in the cell nucleus or cytoplasm, leading to altered gene expression and/or protein function (66, 68, 69). In contrast to canonical biogenesis of miRs, emerging data supports the findings that miRs can also be stably found in a variety of body fluids, such as saliva (70, 71), urine (72), and blood (73, 74) either packaged within exosomes or in complexes with RNA-binding proteins such as Ago or high-density lipoprotein. These miRs, referred to as cell-free miRs (75), may serve as biomarkers of disease (71, 72, 74) and/or facilitators of disease pathogenesis through cell-to-cell communication (75–78).

**Figure 1:**
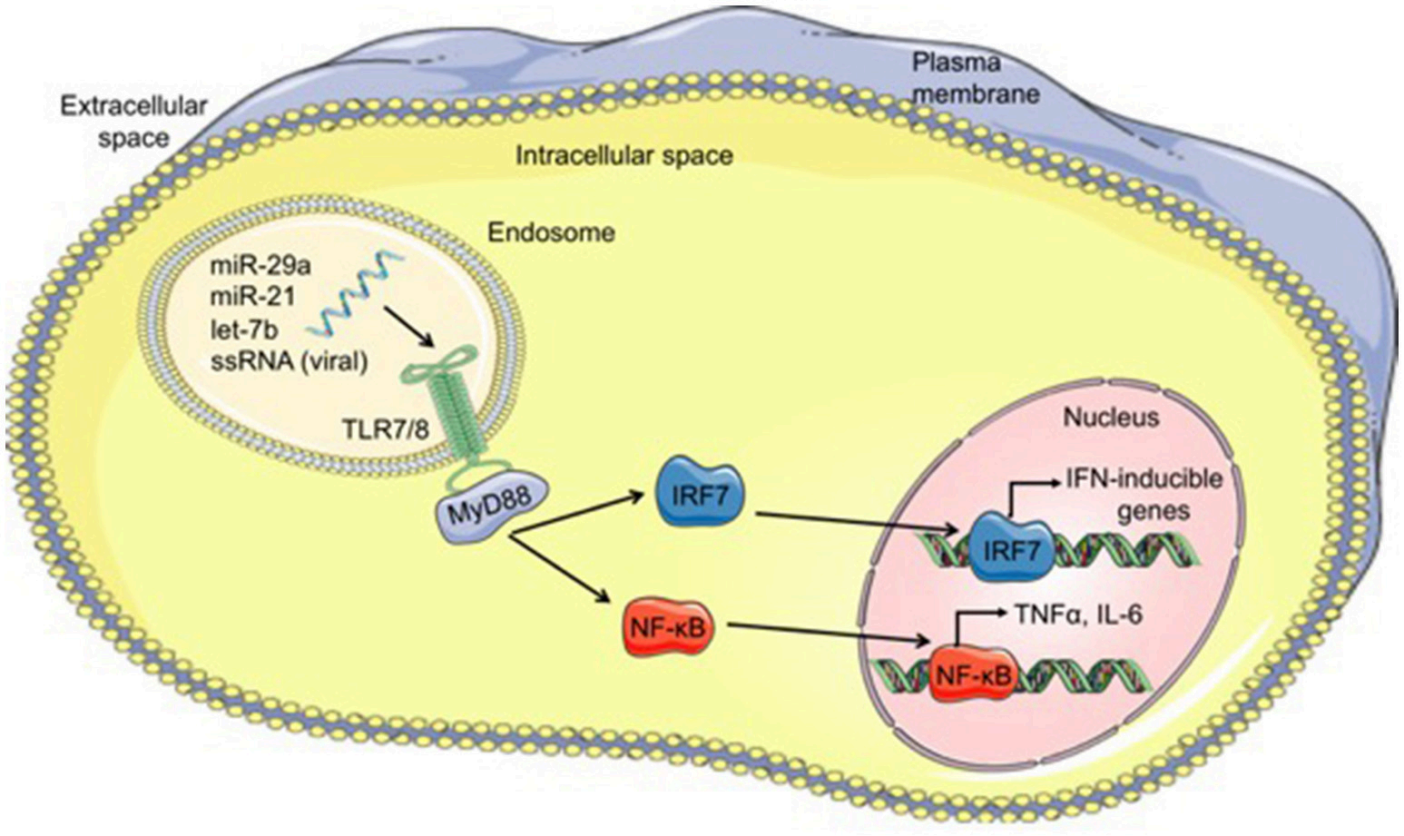
Mechanism of microRNAs functioning as TLR ligands. Cell-derived exosomes containing microRNAs such as let-7b, miR-21, and miR-29a, are taken into cells through endocytosis and fuse with TLR-containing endosomes within cells. The GU-rich microRNA bind to TLR7/8, activating TLR signaling through MyD88 and leading to translocation of IRF7 and NF-κB from the cytoplasm into the nucleus. Once in the nucleus, these transcription factors bind to DNA, resulting in transcription of interferon (IFN)-inducible genes and proinflammatory cytokines such as TNFα and IL-6, respectively. MicroRNA (miR), single-stranded RNA (ssRNA), Toll-like receptor 7/8 (TLR7/8), myeloid differentiation primary response 88 (MyD88), interferon regulatory factor 7 (IRF7), nuclear factor kappa-light-chain-enhancer of activated B cells (NF-κB).

Viral single-stranded RNA oligonucleotides serve as the primary PAMPs recognized by TLR7 (mice) and TLR8 (humans) located within endosomes (79). Since miRs are short single-stranded RNA molecules, it is conceivable that miRs could function as ligands for these specific TLRs. Indeed, over the past 7 years, a small number miRs have been shown to function as TLR ligands in a variety of diseases, which will be discussed briefly. A summary of the mechanism by which miRs can function as TLR ligands is shown in Figure 1.

In 2012, Lehmann et al. demonstrated that secreted miR let-7b functions as a TLR7 ligand in murine microglia and bone marrow-derived, leading to the induction of TNFα release and subsequent neurodegeneration (80). The authors demonstrate that all let-7 family members, not just let-7b, are able to activate TLR7 on murine microglia and propose that this is due to the presence of a conserved 3' GU-rich motif on all let-7 family members that is also present on a known TLR7 ligand, HIV ssRNA40 (79). Interestingly, let-7b was shown to be released from dying neurons *in vitro*, which then functioned in a paracrine manner to accelerate neuronal injury of surrounding neurons. The neuronal damage caused by let-7b release from surrounding degenerative neurons could be ameliorated by pre-treatment with a let-7b inhibitor both *in vitro* and *in vivo* when administered intrathecally. Lastly, the authors also showed elevated let-7b levels in the cerebrospinal fluid of patients with Alzheimer's disease when compared to those who did not have disease. Taken together, these findings demonstrate that let-7b serves as a novel TLR7 ligand on murine microglia, functions as a DAMP to surrounding neurons during times of neuronal injury and could be a novel therapeutic target to reduce neuronal damage.

Kim et al. investigated the role of synovial fluid let-7b in the pathogenesis of an autoimmune disease; rheumatoid arthritis (81). First, the authors identified that let-7b is markedly upregulated in synovial fluid of patients with rheumatoid arthritis. Similar to Lehmann et al., Kim et al. identified that let-7b functions as an endogenous ligand for both TLR7 and TLR8 within synovial fluid macrophages in patients with rheumatoid arthritis. Additionally, let-7b strongly stimulated TLR7-positive myeloid cells found within synovial fluid, driving their development toward pro-inflammatory M1 macrophages in a murine model of rheumatoid arthritis.

In 2012, Fabbri et al. showed that miR-21 and miR-29a are both secreted from lung cancer cell lines into cell-derived exosomes and interact with TLR-containing endosomes within macrophages at the interface between neoplastic and non-neoplastic tissues (82). Once within macrophage endosomes, miR-21, and miR-29a function as TLR ligands to activate murine TLR7 and human TLR8. In contrast to Lehmann et al. which show indirect interaction between let-7b and murine TLR7, Fabbri et al. utilized co-immunoprecipitation assays to demonstrate the direct binding of miR-21 and miR-29a to TLR7/TLR8. Functionally, the binding of miR-21 and miR-29a to TLR8 on human peripheral blood mononuclear cells induces NF-κB-dependent production of both TNFα and IL-6. Similar to let-7b and TLR7, specific short GU-rich motifs found on miR-21 and miR-29a are critical for modulating their binding to and activation of TLR8. Utilizing the inflammation-induced Lewis lung cancer mouse model, the authors demonstrate that tumor-secreted exosomal miRs, including miR-21 and miR-29a, induce murine TLR7 activation and increase the formation of lung multiplicities. Furthermore, treatment of mice with a locked nucleic acid (LNA) antimiR-21/29a significantly reduced pulmonary tumor multiplicities. Altogether, these findings directly demonstrate that miRs can function as TLR ligands, are important regulators of prometastatic inflammation, and warrant additional investigation as novel cancer therapeutic targets.

## MiR29a as TLR ligand in aGVHD

While there are many studies which describe the importance of miRs in aGVHD pathogenesis, the focus is primarily on intrinsic miR expression in immune cells such as T cells and dendritic cells (83–86). Recent data, however, suggests that miRs in circulation may serve as important modulators of pathogenesis in aGVHD.

Our group demonstrated the a novel role for serum miR-29a in aGVHD pathogenesis as a ligand for TLR7/ TLR8 on dendritic cells following allo-HSCT (84). Using two independent cohorts of patients who received allo-HSCT, we showed that miR-29a is upregulated in the serum of patients who develop aGVHD when compared to those who do not. These findings were also validated in murine models of aGVHD and it was further shown that the miR-29a was localized within serum exosomes. Using liposomal-conjugated miR-29a, we showed that murine bone marrow derived dendritic cells (BMDCs) were potently activated, as indicated by upregulation of maturation markers CD40, CD80, CD86, MHC II, and CCR7 as well as significant secretion of pro-inflammatory TNFα and IL-6. BMDCs activated by miR-29a migrated more efficiently toward CCR7 ligand CCL19 and induced stronger T cell proliferation when compared to BMDCs treated with miR-16 which served as a negative control. Because TLR signaling results in activation of My-D88-dependent transcription factors including NF-κB and IRF7, we also showed that miR-29a stimulation of murine BMDCs leads to nuclear translocation of both phosphorylated IRF7 and NF-κB-p65. Lastly, utilizing healthy donor human peripheral blood mononuclear cells (PBMCs) and monocyte-derived DCs, we demonstrated that exosomal miR-29a activates human PBMCs and DCs. To conclusively show that these findings were due to direct binding of miR-29a to human TLR8, Flag antibody tagging, and RNA immunoprecipitation were performed and confirmed marked enrichment of miR-29a in TLR8 transfected DCs only. Finally, we showed that administration of LNA antimiR-29a resulted in reduced circulating serum miR-29a, significantly improved survival and decreased clinical aGVHD severity while maintaining beneficial graft-vs.-leukemia effects in murine models of aGVHD. These findings are the first and only to demonstrate miRs as TLR ligands in the context of aGVHD and provide an exciting novel therapeutic target to prevent or treat aGVHD.

## Future direction

Allo-HSCT remains a curative modality a variety of diseases, including hematologic malignancies, myelodysplastic disorders, myeloproliferative neoplasms, and aplastic anemia (3, 87, 88). Despite this, aGVHD remains a frequent and lethal complication of allo-HSCT, underscoring the need for better understanding of aGVHD pathogenesis Furthermore, given the high morbidity and mortality associated with aGVHD, scientists and clinicians are seeking not just novel therapeutics but also novel prophylaxis. TLRs are highly conserved PRRs which are a critical aspect of the innate immune system. Traditionally, TLRs are activated by DAMPs and PAMPs, many of which are involved in the initiation of aGVHD. MiRs, being small ssRNA strands, have recently been documented as serving as ligands for TLR7 and TLR8, propagating such diseases as Alzheimer's disease, rheumatoid arthritis, and aGVHD. With the identification of miR-29a as a soluble mediator of TLR activation in aGVHD, many additional questions arise. Are there other secreted miRs which activate TLRs in the context of aGVHD? While miR-153-3p has recently been identified as a plasma miR which is upregulated and disrupts tryptophan synthesis during aGVHD, the authors do not evaluate the role of miR-153-3p on specific immune cell activation such as T cells or APCs (78). Secondly, do these miRs interact with their associated TLR similarly with specificity dependent on GU-rich motifs and how much specificity does this confer? As miRs are < 25 nt long and thousands of miRs are currently recognized, the potential for other miRs to have similar GU-rich motifs which could bind to TLRs seems highly likely. Third, can these miRs serve as novel therapeutic or prophylactic targets in diseases in which TLR7/8 play a pivotal role in pathogenesis, including aGVHD? TLR agonists and antagonists are actively being investigated as novel therapeutics for a broad range of diseases, including cancer, inflammatory disease, allergies, and infectious agents such as HIV and hepatitis C (89–92), although TLR modulation as an aGVHD therapeutic is still very much in its infancy. With this, we have the potential for gaining a better understanding aGVHD pathogenesis and identifying novel prophylactic and therapeutic targets. With all of these questions still needing answers, we have likely only brushed the surface, opening a completely new avenue of study for scientists not only in aGVHD but across many diseases in which TLR7/8 is implicated in their initiation and development.

## Author contributions

All authors listed have made a substantial, direct and intellectual contribution to the work, and approved it for publication. NZ and PR wrote the manuscript together. RG edited the manuscript.

### Conflict of interest statement

The authors declare that the research was conducted in the absence of any commercial or financial relationships that could be construed as a potential conflict of interest.
